# Spontaneous formation of gold nanostructures in aqueous microdroplets

**DOI:** 10.1038/s41467-018-04023-z

**Published:** 2018-04-19

**Authors:** Jae Kyoo Lee, Devleena Samanta, Hong Gil Nam, Richard N. Zare

**Affiliations:** 1 Department of Chemistry, Stanford University, Stanford, CA 94305 USA; 2 Center of Plant Aging Research, Institute for Basic Science, Daegu, 42988 Republic of Korea; 3 Department of New Biology, DGIST, Daegu, 42988 Republic of Korea

## Abstract

The synthesis of gold nanostructures has received widespread attention owing to many important applications. We report the accelerated synthesis of gold nanoparticles (AuNPs), as well as the reducing-agent-free and template-free synthesis of gold nanoparticles and nanowires in aerosol microdroplets. At first, the AuNP synthesis are carried out by fusing two aqueous microdroplet streams containing chloroauric acid and sodium borohydride. The AuNPs (~7 nm in diameter) are produced within 60 µs at the rate of 0.24 nm µs^−1^. Compared to bulk solution, microdroplets enhance the size and the growth rate of AuNPs by factors of about 2.1 and 1.2 × 10^5^, respectively. Later, we find that gold nanoparticles and nanowires (~7 nm wide and >2000 nm long) are also formed in microdroplets in the absence of any added reducing agent, template, or externally applied charge. Thus, water microdroplets not only accelerate the synthesis of AuNPs by orders of magnitude, but they also cause spontaneous formation of gold nanostructures.

## Abstract (web summary)

Reactions in aqueous microdroplets can significantly differ from those in bulk. Here, the authors report microdroplets that not only accelerate gold nanoparticle formation by several orders of magnitude but also promote spontaneous nanostructure formation with no reducing agents or template.

## Introduction

Gold nanostructures are one of the most widely studied materials owing to the unique properties of nanoscale gold such as quantum size effects, surface plasmon resonance, high catalytic activity, self-assembly, etc.^1–5^. The synthesis of gold nanoparticles (AuNPs) typically takes seconds to hours^6,7^. In many cases, capping agents are added because they stabilize the nanoparticles and also act as structure-directing agents, leading to the formation of anisotropic nanostructures such as gold nanowires (AuNWs), nanoplates, nanoprisms, etc.^8,9^. Alternatively, the templates can be used for the anisotropic growth of gold nanostructures^10–12^. Control over both size and shape of the nanostructures is important as both factors strongly influence optical, mechanical, electrical, and chemical properties of the nanostructures.

Recently, microdroplet chemistry has emerged as a unique platform for studying and performing chemical reactions. Microdroplet fusion mass spectrometry, developed by our group previously, allows the capture of events occurring on the microsecond timescale^13^. It has been found that chemical reactions in micron-sized liquid droplets (microdroplets) exhibit unusual reaction properties that are not observed in bulk solutions; in particular, some reaction rates can be enhanced by factors of 10^3^–10^6^ ^13–27^. All reactions explored to date are simple bimolecular reactions. We report the crystallization kinetics of gold precursors in the microdroplets. Our objective was to study the formation and growth of AuNPs in microdroplets and whether nanocrystallization in the microdroplets is different from that in the bulk solution. Accelerated crystallization and subsequent nanoparticle formation are fundamentally different from bimolecular reactions, as it requires fast reaction and coalescence of hundreds of individual species within a span of microseconds.

There have been several studies on nanoparticle synthesis using micron-sized reactors (e.g., using microfluidic platforms), mostly aimed at precise manipulation of growth, rapid mixing, fast time-resolved analysis, and multiple phase synthesis^28,29^. However, the controllable timescale for growth or analysis using the microreactors is only limited to tens of milliseconds with sub-second dead times^30^. Another approach using microreactors for nanoparticle synthesis is spraying the charged droplets containing precursor metal ions onto a grounded cathode surface, in which the metal nanoparticles are formed on the cathode surface^31^. This study did not provide information on the kinetics of the process.

In contrast to these previous approaches, we used high-speed fused aerosol microdroplets for nanoparticle formation, allowing observation with microsecond temporal resolution. We followed a well-established procedure, where AuCl_4_^−^ ions coming from chloroauric acid (HAuCl_4_) are reduced by sodium borohydride (NaBH_4_)^32,33^. NaBH_4_ is a classic reductant that plays dual roles of reducing gold ions and stabilizing AuNPs against aggregation^32^.

We investigate the kinetics of AuNP formation by fusing the two microdroplets, each containing one reactant, and varying the traveling distance of the fused microdroplets to the collection device. As a control, we also examine what happens when we use aqueous microdroplets containing no added reducing agent. We find that the microdroplets are also effective in producing AuNPs and AuNWs in the absence of any reducing agent and template. We also find that this occurred in microdroplets without any added reducing agents or externally applied charges. The spontaneous formation of nanostructures is attributed to the entropic changes that overcome the free-energy barrier in the reaction, caused in part by a strong electric field at the water–air interface of the microdroplets. Our results further reveal the unique properties of the microdroplets and provide a new method for a highly efficient synthesis of nanostructures under benign, environmentally friendly, and ambient conditions.

## Results

### Kinetically controlled growth of AuNPs in microdroplets

**Fig. 1:**
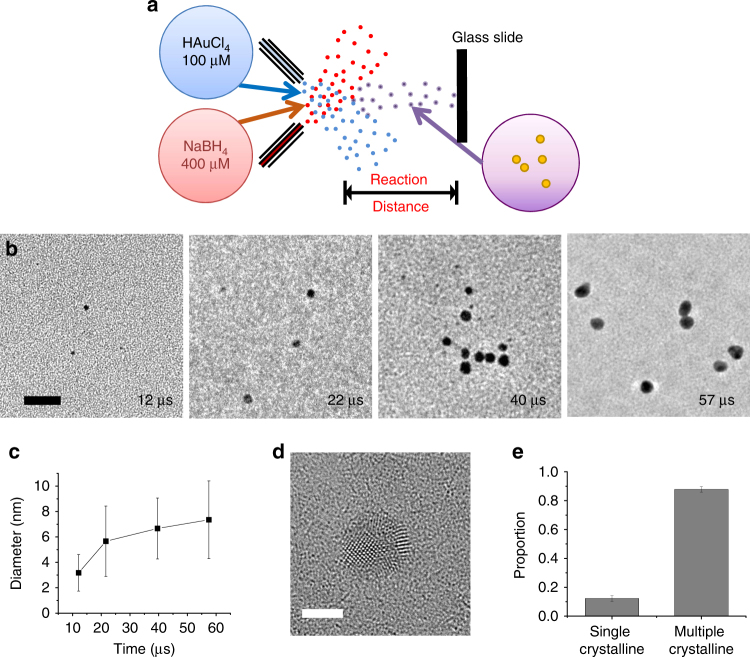
Kinetically controlled AuNP growth using fused microdroplets. **a** Schematic of the experiment setup. Microdroplets containing 100 µM HAuCl_4_ solution were fused with the ones containing 400 µM NaBH_4_ solution. The crystallization process was kinetically controlled on the microsecond timescale by adjusting the traveling distance of the fused microdroplets. **b** TEM images of gold nanoparticles at different time points. Scale bar is 20 nm. **c** Time-course change in the diameter of the gold nanoparticles. **d** TEM image of a nanoparticle showing multiple crystalline structures. **e** Portion of nanoparticles with single or multiple crystalline structures synthesized in microdroplets. Error bars represent one standard deviation of three replicates

Figure 1a shows a schematic of the experimental setup. A stream of aqueous microdroplets containing 100 µM HAuCl_4_ solution was fused with a stream of aqueous microdroplets containing 400 µM NaBH_4_ solution. Unlike previous studies with fused microdroplets, no voltage was applied to either of the streams. The reaction progressed as the fused microdroplets traveled toward a glass slide where they were collected. The reaction time was adjusted by varying the traveling distance. The estimated reaction time was calculated by integrating the speed of the microdroplets over the traveling distance, as previously described^13^. The size of a fused aqueous microdroplet remained almost constant within the observation time frame, showing that almost minimal evaporation occurred in the microdroplets during their traveling time^13,15^.

Figure 1b shows representative transmission electron microscope (TEM) images of the synthesized AuNPs at different reaction times (12–57 µs). Exposure time and intensity of the e-beam for TEM imaging were minimized to avoid sintering or modification of the nanoparticle structure. Figure 1c presents the time-course change in the averaged diameter of AuNPs. We observe that within as early as 12 µs, particles as large as 3 nm are formed. The rate of the nanoparticle growth was calculated to be 0.24 nm µs^−1^. The reaction notably slowed down after ~21 µs, although the reaction did not reach equilibrium within the observation time frame. The mean diameter of nanoparticles at 57 µs was ~7 nm. Our results indicate that nanoparticle formation involves fast nucleation followed by slower growth. These results are consistent with the mechanism put forward by Emmerling and co-workers, who measured the growth rate of AuNPs by mixing ~500 µM HAuCl_4_ and 2 mM NaBH_4_ solutions, flowing the mixed solution in a 1-mm size channel, and monitoring the growth kinetics with on-line small-angle X-ray scattering^34^. They observed that nucleation occurred within 200 ms, the resolution limit of their set up. The growth rate of nanoparticles in the bulk solution was ~0.03 nm s^−1^. It took several hours for the reaction to reach equilibrium. In comparison, we find that in microdroplets, the initial nucleation events occur at least 2.8 × 10^4^ fold faster. Moreover, the growth of AuNPs in the microdroplets was enhanced by a factor of 1.2 × 10^5^, compared to bulk solution, even considering the higher concentrations of precursors used for the reaction in bulk continuous flow. Comparing the size of AuNPs between the microdroplet and bulk solution after the reaction significantly slowed down, the AuNP size in the microdroplet was ~2.1 times larger than that in the bulk solution. A high-resolution TEM image (Supplementary Fig. 1) shows the indexing of the two-dimensional lattice fringes to be ~2.3 Å, corresponding to the lattice spacing of Au (111). This confirms that the nanoparticles have the crystal structure of pure Au.

Figure 1d, e shows a TEM image of a representative nanoparticle with multiple crystalline structure and the relative proportion of single and multiple crystalline structures. It is not clear whether the multiple crystalline structures were formed by smaller single crystalline nanoparticles coalescing or by the inhomogeneous environment of the microdroplet. Further kinetic studies at higher temporal resolution with a slower reaction rate, for example, by lowering the growth temperature or the precursor concentration, may reveal what mechanism is responsible for the multiple crystalline structures. To date, we have not succeeded in sufficiently slowing down the crystallization process to acquire this information.

We confirmed that most of the AuNP growth occurred in microdroplets rather than on the surface of the glass slide or during the transfer to a TEM grid. This was achieved by examining the average diameter of nanoparticles under two different drying conditions. The size of AuNPs prepared by air drying for 30 min after collecting the aqueous microdroplets containing nanoparticles are not significantly different from the size of the nanoparticles prepared in a vacuum desiccator for 5 min (Supplementary Fig. 2).

### AuNP formation in reducing-agent-free microdroplets

**Fig. 2:**
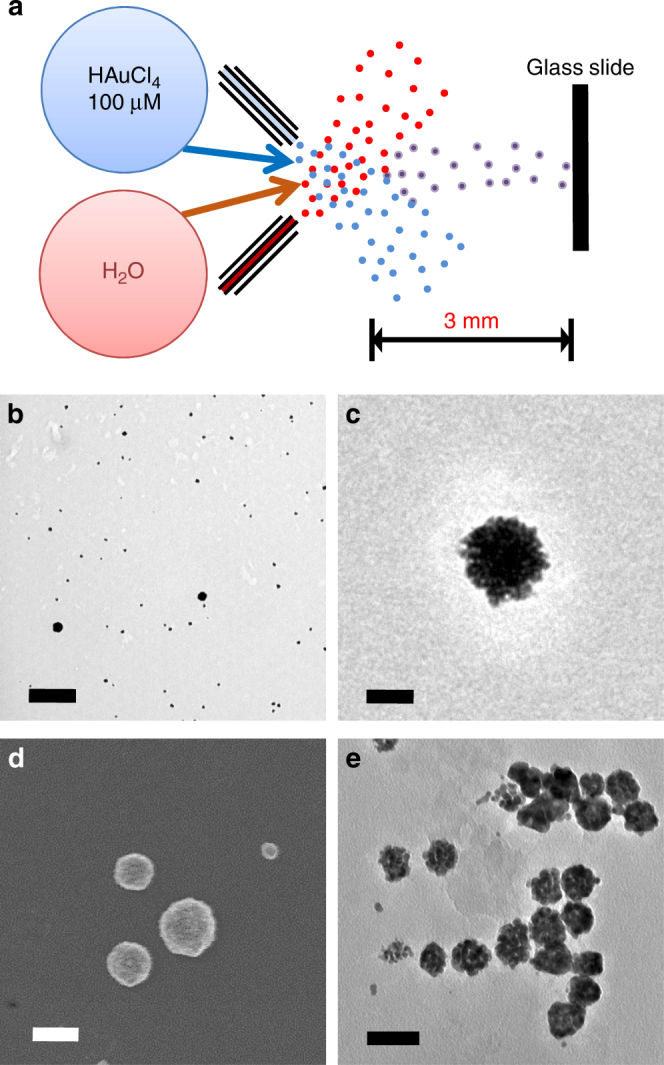
Reducing-agent-free synthesis of AuNPs in the microdroplets with no externally applied charges. **a** schematic of the experimental setup for reducing-agent-free synthesis of AuNP in the microdroplets. Scale bar is 1 µm. **b**, **c** TEM images of the synthesized AuNPs and AuNP aggregates. **d** SEM image of the AuNP aggregates. **e** TEM image showing an intermediate aggregated status of AuNP aggregates. Scale bars for **b**–**e** are 50 nm

As a control experiment, we fused the aqueous droplets containing 100 µM HAuCl_4_ with the aqueous microdroplets (Fig. 2a) without any externally added charges. The fused microdroplets containing only HAuCl_4_ solution were collected after 3 mm of traveling for ~40 µs traveling time. Surprisingly, we have found the formation of AuNPs, as evidenced by the TEM image in Fig. 2b. Closer inspection revealed that in addition to small nanoparticles (~7 nm in diameter), larger particles (~30 nm in diameter), caused by non-specific aggregation of the AuNPs, were also formed (Fig. 2c). The identity of the AuNPs was verified by energy dispersive spectroscopy (EDS) (Supplementary Fig. 3) as well as by measuring the lattice spacing. A high-resolution SEM image confirmed that the shapes of the AuNP particles are spherical (Fig. 2d). Figure 2e presents a TEM image showing both individual AuNPs and AuNP aggregates, as well as half-way aggregated intermediate forms that illustrates how the AuNP aggregates were formed.

We examined whether inelastic collisions of the microdroplets, which provide kinetic energy, may contribute to the nanoparticle formation. We used only one stream of microdroplet containing 100 µM HAuCl_4_ solution without fusing with other microdroplets. The microdroplets traveling for 3 mm were collected on a glass slide. Similar nanoparticle or nanoparticle-aggregate formation was observed using single stream of microdroplets, suggesting that the nanoparticle formation in the microdroplets was not caused by collisional activation. The nanoparticle formation was further verified by collecting the HAuCl_4_ microdroplets at 3 mm distance from the stream over a period of 30 min and carrying out dynamic light scattering analysis.

We also ruled out the possibility of unintended formation of AuNPs by the e-beam irradiation used for TEM imaging, as the e-beam can produce and manipulate metal nanoparticles^35–37^. Supplementary Figure 4 shows two TEM images taken before and after a continuous e-beam exposure for 20 min. No difference in the structure of the nanoparticles or no new formation of nanoparticles or any other nanostructure confirms that the nanoparticles were not synthesized by the e-beam used for the TEM imaging, rather the effect of microdroplets. We also ascertained that AuNPs were not formed through the physical contact with metal substrates in the TEM grids by examining the TEM grids made from different materials.

To explore the mechanism of the AuNP formation in the absence of any reducing agent, we carried out mass spectrometric analysis of HAuCl_4_ solution in the microdroplets. A 10 µM HAuCl_4_ solution was sprayed in forms of microdroplets. The travel distance of microdroplets was 1.5 cm that allows the analytes to be exposed to the microdroplet environment for ~180 µs. Supplementary Figure 5 shows a mass spectrum of the HAuCl_4_ solution in the microdroplets. The original form of gold, AuCl_4_^−^, with an oxidation number + 3, as well as the reduced forms of gold, Au_2_Cl_3_^−^ (oxidation number + 1), AuCl_2_^−^ (oxidation number + 1), and Au_2_Cl_4_^−^ (oxidation number + 1.5) were observed. Supplementary Table 1 summaries the gold molecular species observed in the microdroplets containing HAuCl_4_ solution. We also examined the possibility of thermal decomposition of AuCl_4_^−^ ions^38^ that can be possibly caused due to the high temperature at the mass spectrometer capillary inlet (275 °C). We have decreased the temperature of the capillary inlet down to 60 °C, below the typical thermal decomposition temperature. Essentially, the same mass spectrum was acquired with spontaneous reduction of gold ions at the lowered temperature. These results present direct evidence that gold ions were reduced spontaneously to form AuNPs in the microdroplets without an added reducing agent.

### AuNW growth in droplets free of reducing agent and template

**Fig. 3:**
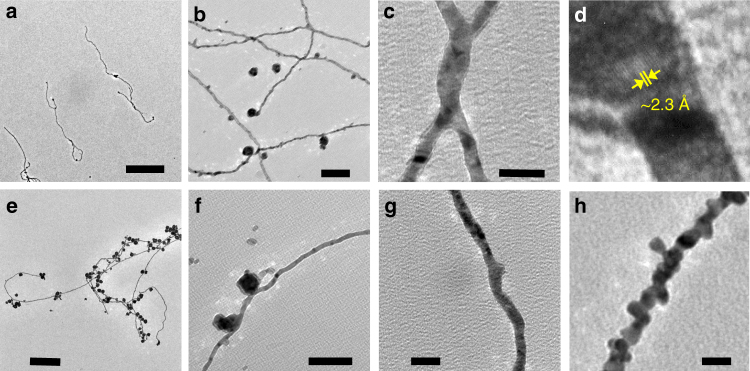
Reducing-agent-free and template-free synthesis of Au nanowires (AuNWs) in the aqueous microdroplets. **a** AuNWs with several micrometer length. Scale bar is 1 µm. **b** Formation of AuNW network. Scale bar is 100 nm. **c** Junction between two AuNWs. Scale bar is 20 nm. **d** A TEM image showing the lattice spacing of ~2.3 Å, corresponding to the Au(111) structure, confirming that the nanowire is made of pure Au. **e**,** f** AuNWs accompanying with AuNP and AuNP aggregates. Scale bars for **e** and **f** are 300 nm and 100 nm. **g**,** h** Two different types of AuNWs formed in the microdroplets with a smooth surface (**g**) and beaded surface (**h**). Scale bars for **g** and **h** are 20 nm

AuNWs can be synthesized by various methods including chemical, electrochemical, and epitaxial routes^39,40^. The formation often needs structure-directing capping agents^41^ or porous templates that guide their directional growth^42^. However, we find that AuNWs (~7 nm wide) can be formed in the microdroplets without using any template or reducing agent, as well as externally applied charges (Fig. 3). The average length of AuNWs formed in aqueous microdroplets was 2195 nm, raging from hundreds of nanometer to approximately six micrometers (Fig. 3a). Figure 3b, c shows that not only single-strand wires, but also a network of nanowires appeared. The constitution of the nanowires with pure Au was confirmed by the lattice spacing of ~2.3 Å, matching with the Au (111) structure (Fig. 3d), and EDS analysis (Supplementary Fig. 6).

A form of nanowires accompanied with AuNP or AuNP aggregates were also observed (Fig. 3e, f). This nanowire and nanoparticle junction can potentially be used for remote excitation of surface-enhanced Raman scattering^43^, catalysis, or physical entrapment of materials^44^. Two different forms of AuNWs were found in the microdroplets: one with a relatively smooth surface (Fig. 3g) and the other with a beaded surface (Fig. 3h). The beaded wires appears to be composed of a series of individual AuNPs. On the other hand, the nanowires with smooth surface do not appear to be made of individual or clusters of AuNPs, although there are multiple crystalline domains present.

### Kinetics of spontaneous AuNP/AuNW growth in the microdroplets

**Fig. 4:**
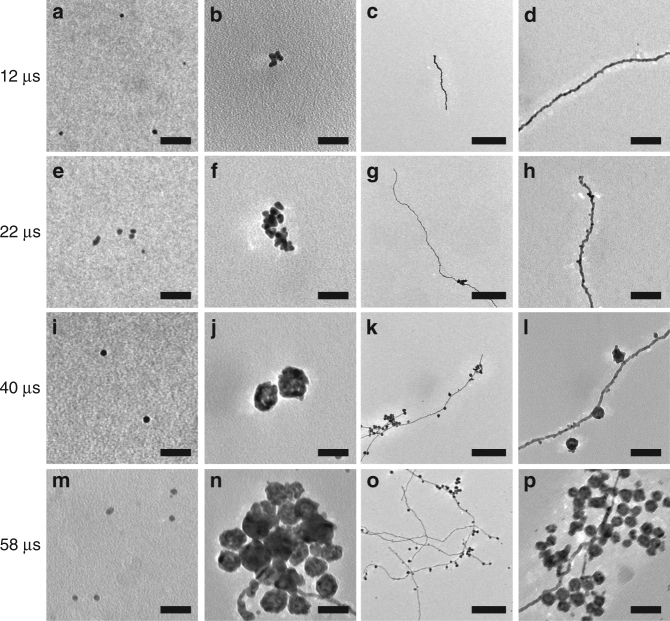
TEM images at different time points of AuNPs and AuNWs grown in the aqueous microdroplets. Time-course changes in the individual AuNPs (**a**,** e**, **i**,** m**), AuNP aggregates (**b**,** f**,** j**,** n**), and AuNWs (**c**,** d**,** g**,** h**,** k**,** l**,** o**,** p**). Scale bars for **a**,** b**,** e**,** f**, **i**,** j**, **m**, **n** are 40 nm. Scale bars for **c**,** g**,** k**,** o** are 80 nm. Scale bars for **d**, **h**,** l**,** p** are 80 nm

**Fig. 5:**
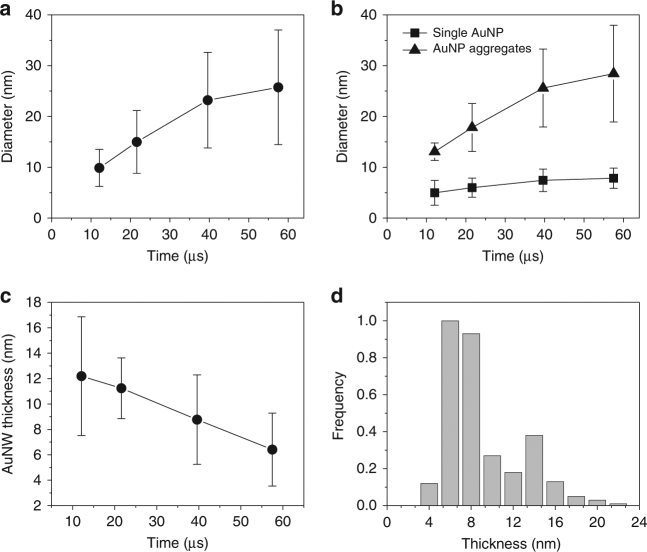
Kinetics of AuNPs and AuNWs growth in the reducing-agent- and template-free microdroplets. **a** The diameter of AuNP aggregates as a function of time in the microdroplets. **b** The change in the diameters of individual AuNP and AuNP aggregates over time. **c** The change in the thickness of AuNW over time. **d** The bimodal distribution of the thickness of AuNWs formed in the microdroplets. Error bar represents standard deviation of the three replicates

We further studied the kinetics of the spontaneous AuNP and AuNW growth in the microdroplets. Aqueous microdroplets containing 100 µM HAuCl_4_ solution were generated using high-pressure nebulizing dried N_2_ gas. The traveling distance between the point where the microdroplets were generated and the point where the microdroplets were collected was adjusted to control the exposure time of Au precursors to the microdroplet environment. The collected samples were transferred to a TEM for imaging analysis. Figure 4 shows the TEM time-series images of individual AuNP growth (Fig. 4a, e, i, m), AuNP aggregates (Fig. 4b, f, j, n), and AuNWs (Fig. 4c, d, g, h, k, l, o, p). Figure 5a–c shows the quantitative analysis of the time-course changes in overall AuNP diameter (Fig. 5a), individual AuNP and AuNP aggregates (Fig. 5b), and the thickness of AuNW (Fig. 5c). Although the overall size of AuNP including individual AuNP and AuNP aggregates, increases (Fig. 5a), the size of an individual AuNP does not grow significantly (only up to ~ 8 nm, see Fig. 4a, e, i, m, and Fig. 5b). The increase of average AuNP size was mainly attributed to AuNP aggregates rather than the increase in individual AuNP size.

Interestingly, the thickness of AuNW decreased over time in the microdroplets, which was somewhat counterintuitive. There were two different types of AuNWs: the one with a smooth surface and the other with a beaded surface. The bimodal distribution of the thickness of AuNWs grown in the microdroplets is reflective of the size difference between the two types of AuNWs (Fig. 5d). The thickness of the smooth-surfaced AuNWs is centered at around 6 nm, whereas the beaded AuNWs at around 14 nm. The decrease in the thickness of the AuNW was because the portion of beaded AuNWs became smaller than that of the smooth AuNWs. It is still unclear what causes the relative proportion of the nanostructures to vary with time.

We have carried out experiments of spraying the fused microdroplets on a NaBH_4_-deposited glass slide to examine whether any further reaction can occur during the collection on a glass slide. Two separate types of experiments: (1) the fused microdroplets between the microdroplets containing 100 µM HAuCl_4_ and the microdroplets containing 400 µM NaBH_4_, and (2) the fused microdroplets between the microdroplets containing 100 µM HAuCl_4_ and the aqueous microdroplets with no NaBH_4_. The traveling distance of the fused microdroplets to the NaBH_4_-desposited collector was 15 mm. For the fused microdroplets containing both HAuCl_4_ and NaBH_4_, there was essentially no difference in the distribution of size of the synthesized nanoparticles (Supplementary Fig. 7). This suggests that the process of nanoparticle formation is almost completed in the microdroplets before reaching the collection slide. On the other hand, for the fused microdroplets between only HAuCl_4_ and water microdroplets, more nanoparticles with <10 nm diameter were observed in the sample collected on the NaBH_4_-desposited slide. This suggests that there were remaining gold ions that were not completely reduced during the travel to the collector in the reducing-agent-free aqueous microdroplets. The remaining gold ions were reduced and formed observed nanoparticles with <10 nm diameter in a thin liquid layer with approximately several hundreds of micrometers thickness containing dissolved NaBH_4_ from the deposited NaBH_4_ on the collector. The surface effect of microdroplets would be much stronger than that of the thin liquid layer on the collector, given approximately the two order of magnitude higher surface-to-volume ratio of microdroplets compared to that of liquid layer. However, it should be noted that further reactions on the collector cannot be eliminated unless the liquid in the solution containing the unreacted gold ions is immediately desolvated upon arriving at the collector.

The separation between the three different nanostructures of nanoparticles, aggregates, and nanowires can be kinetically controlled. We have added Supplementary Fig. 9 showing the changes in the proportion of the three different types of nanostructures as a function of reaction time. At a short time of around 7.5 µs, the majority of the nanostructures synthesized in the microdroplets were individual nanoparticles. At 12.1 µs, the aggregates of individual nanoparticles and nanowires started forming. The proportion of nanowire gradually increased, while the nanoparticle aggregates reached a peak at 12.1 µs and decreased. This data suggest that different types of nanostructures are formed at different temporal points; therefore, different types of gold nanostructures can be collected by controlling the microdroplet traveling time.

## Discussion

We have observed that the formation of AuNPs in the microdroplets accelerated by five orders of magnitude compared to bulk, when HAuCl_4_ and NaBH_4_ are used as precursors. We also found that AuNPs are formed spontaneously from spraying an aqueous solution of HAuCl_4_ in the absence of a reducing agent. In addition, the nanowires and the nanowire networks are formed in these aqueous microdroplets without the use of templates or capping agents, as well as externally applied charges.

We found that by fusing streams of HAuCl_4_ and NaBH_4_ particles as large as ~3 nm were formed only within 12 µs, indicating that nucleation events occur within this timescale. The particles grew up to ~7 nm in diameter within 60 µs. The formation of AuNPs follow fast nucleation and slower growth mechanism, as observed in bulk by Emmerling and co-workers^34^. In contrast to the bulk reaction, the growth rate is increased by a factor of 1.2 × 10^5^ and the equilibrium particle diameter is 2.1 times higher, although the concentration of the reagents used in the present study is five times less.

The accelerated formation of AuNPs in the microdroplets is consistent with other reports of reaction acceleration and catalytic behavior of microdroplets published in the last decade by us, as well as other groups^13–16,19–25^. However, this is the first report on accelerated crystallization in the microdroplets, which is fundamentally different from the bimolecular reactions studied previously. We speculate that the mechanism of enhanced reaction rate in the microdroplets stems from a combination of factors, mostly from air–water interface effects: surface localization of reactants, facilitated diffusion, self-alignment or organization of reactants, inhomogeneous distribution of dissociated ions, and the strong electric field at the surface of the microdroplet^27,45,46^. These combined effects might accelerate the AuNP formation in the microdroplets by either increasing the collision frequency of the precursors or lowering the entropic barriers of reactions.

It is surprising that AuNPs are also formed in the microdroplets without the use of any reducing agent or externally applied charge. Although we observe individual nanoparticles, we also observe aggregates, which can be attributed to the absence of a stabilizing agents. From the kinetic data presented in Figs. 4 and 5, it can be seen that gradual aggregation of the nanoparticles occurs with progression in time. However, it seems the aggregates reach a stable size of ~30 nm in diameter. We are not able to elucidate completely the mechanism of AuNP formation under these conditions, although some characterization has been made in the present studies. We confirmed the reduction of gold ions at molecular level by examining the mass spectrum of HAuCl_4_ solutions in the microdroplets. The gold ions with reduced oxidation number of +1 and +1.5 were observed, compared to the original oxidation number of +3 for AuCl_4_ ion. Further details on the mechanism of the spontaneous reduction are being investigated currently in our laboratory and will be presented in the follow-up publication.

Prior studies have shown that electric fields as high as 8.9 × 10^7^ V m^−1^ exist across the air–water interface^47^. It has been reported that ions are distributed nonuniformly throughout the aqueous microdroplets. The electric field strength can be much enhanced if the charge separation distance reduces. This electric field strength can be further increased by fluctuations occurring at the microdroplet surface, which can be induced by air–water friction or capillary waves^48^. These large electric fields may promote the reduction of AuCl_4_^−^ to Au. Alternatively, it has also been shown that the surface of water is rich in hydroxyl ions. Considering the redox potential of AuCl_4_^−^ + 2e^−^ → AuCl_2_^−^ + 2Cl^−^ is +0.926 V and that of O_2_ + H_2_O + 4e^−^ → 4OH^−^ is +0.4 V, it is possible that hydroxyl ions of water are oxidized, reducing AuCl_4_^−^ in the process.

We have performed experiments to capture any oxygen species evolved during the nanostructure formation in the microdroplets. We have used tris(2,2′-bipyridyl) dichlororuthenium (II) cation (Ru(bpy)_3_^2+^), which is water soluble and widely used for capturing the dissolved oxygen in liquids^49,50^. Supplementary Figure 10a shows a mass spectrum of the microdroplets containing 10 nM Ru(bpy)_3_^2+^. Dried N_2_ nebulizing gas was used to minimize the exposure of the microdroplet surface to oxygen in air. A peak of Ru(bpy)_3_^2+^ with Cl^−^ adduct was observed at *m/z* 605.08. To examine whether the exchange of the surrounding gas to O_2_ would affect the oxidation of Ru(bpy)_3_^2+^, the nebulizing gas was changed to O_2_. No oxygenated species were observed after these changes (Supplementary Fig. 10b). Next, we recorded the mass spectrum of the microdroplets containing 10 nM Ru(bpy)_3_^2+^ and 50 µM HAuCl_4_. An oxygenated Ru(bpy)_3_ species was detected at *m/z* 585.10, which was suggested to be Ru(bpy)_2_(bpyO)^51^. All the procedures were conducted in a dark room to prevent any light-induced oxidation or decomposition of reagents. These data show that oxygen evolves during the reduction of gold ions and nanostructure formation in the microdroplets and support our argument that hydroxyl ions of water are oxidized to reduce gold ions. The oxygenated Ru(bpy)_3_ species was observed only when HAuCl_4_ was added to the microdroplets. This is because oxygen evolves through electron transfer only when a participating redox pair is present.

A third possibility is reductive elimination of chlorine gas. However, the reaction AuCl_4_^−^ → AuCl_2_^−^ + Cl_2_ is endergonic (>12.6 kcal mol^−1^) in bulk^52^. It remains to be investigated whether the free-energy change in the microdroplet environment accounts for what we have observed. We have confirmed that for some reactions, the microdroplets do change the free energy of the reaction and facilitate the otherwise unfavorable reactions^53^.

**Fig. 6:**
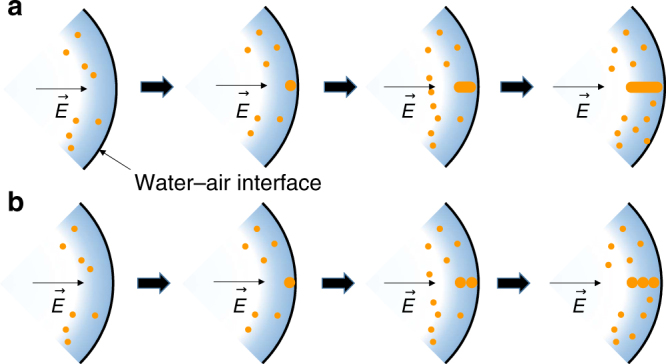
Possible mechanism for nanowire growth in the microdroplets. **a** AuNW formation by the growth of an initial seed at the surface of the microdroplet. **b** AuNW grown by the self-assembly of AuNPs

The electric field also appears to be involved in the nanowire formation in the microdroplets. Figure 6 presents a possible mechanism in which the nanowires can be formed at the water–air interface. The nonuniformly distributed ions can exert a high electric field at the water–air interface. Several studies have reported electric-field-induced nanostrucure alignment or anisotropic growth^54–56^. The electric field that is orthogonal to the microdroplet surface may direct the growth of the Au nanostructure from the microdroplet surface to the center of the microdroplets. As growth of the initial nanowire occurs, the formed nanowire can further increase the charge accumulation at the tip of the nanowire that can promote growth at the tip of the nanowire (Fig. 6a). It is also possible that the nanowire is grown by collision of the nucleus or seed nanoparticles at the terminal of the initially grown nanowire (Fig. 6b). From the Figs. 4 and 5, it can be deduced that initially the AuNWs are grown by the mechanism proposed in Fig. 6b and followed by Fig. 6a. Based on the kinetic data (Figs. 4 and 5), we propose the following mechanism for the formation of AuNWs. We observe that the width of the AuNWs are more or less invariant with time and is similar to the diameter of the AuNPs formed, although the length of the wires increases. From Fig. 4, it is clear that the AuNPs formed a self-assembly to form some AuNWs. Self-assembly of AuNPs into AuNWs have been observed before. For example, Bjørnholm et al. demonstrated the formation of 2D AuNWs at the air–water interface by using surfactants^57^. Rajh et al. have shown the self-assembly of the charged AuNPs on exposure to electron beam^58^. The present work is a striking demonstration of self-assembly of nanoparticles without the use of any surfactants or externally applied strong fields.

We have studied the formation of gold nanostructures in the aqueous microdroplets traveling through air at room temperature. We have found that the reaction rate is enhanced by many orders of magnitude, compared to bulk solution. We have also discovered an unexpected and new phenomenon: spontaneous formation of AuNPs, as well as nanowires in the absence of any reducing agent or template. Although not completely understood, we have suggested three possible mechanisms for this reaction. Our results indicate that the AuNWs are formed by self-assembly of the nanoparticles. It should be noted that this is the first demonstration where self-assembly can be obtained without the use of surfactants or externally applied strong fields. Our results demonstrate that microdroplets have a unique environment and hold the potential to be used as powerful microreactors for the synthesis and template-free control of nanostructures.

## Methods

### Chemicals

Gold(III) chloride trihydrate (HAuCl_4_∙3H_2_O, 520918), NaBH_4_, and tris(2,2-bipyridyl)dichlororuthenium(II) hexahydrate were purchased from Sigma-Aldrich (St. Louis, MO). HPLC-grade water were purchased from Fisher Scientific (Nepean, ON, Canada).

### Synthesis of AuNPs using microdroplet fusion

We used the microdroplet fusion setup for kinetically controlled growth of AuNPs. The microdroplet fusion was conducted as previously described^13^. Briefly, two streams of the microdroplets were generated by a high-pressure, dry N_2_ gas at 120 psi. The two microdroplet sources are equipped with an X–Y–Z micro-positioning linear and angular stage for accurate alignment of the two microdroplet streams. This alignment is important for ensuring fusion of most of the incident droplets and for maintaining a linear trajectory toward the collector. The best alignment was acquired with the angle between two crossed microdroplet streams at 78°, which showed the highest probability of droplet fusion and straight trajectories of the fused microdroplets to the inlet of the mass spectrometer. Two solutions of analytes (HAuCl_4_ solution at 100 µM and NaBH_4_ at 400 µM) were injected from the two microdroplet sources with a syringe pump (Harvard Apparatus, Holliston, MA) at a flow rate of 30 μL min^−1^. The stream of fused microdroplets at a speed of 83 m s^−1^, which was previously measured in the charged microdroplets using a high-speed camera and particle tracking^13^, was collected on glass slides. This speed is assumed to be similar to that of the uncharged microdroplets used in the present studies. This reason is because the predominant factor affecting the velocity of the microdroplets is the nebulizing gas pressure, rather than the Coulomb force between the source of the microdroplets and the counter electrode. The collected fused microdroplets containing reaction products were transferred to a TEM grid for TEM imaging. The samples were dehydrated completely prior to being mounted onto the TEM holder. The sample drying was carried out in open air or in a vacuum desiccator. The approximate drying durations were 30 min in open air and 5 min in a vacuum desiccator.

To examine whether any further reaction can occur during the collection on a glass slide, the experiments of spraying the fused microdroplets on NaBH_4_-deposited glass slides was conducted. The stream of the fused microdroplets generated by fusing the microdroplets containing 100 µM HAuCl_4_ solution in water and the microdroplets containing 400 µM NaBH_4_ solution in water were sprayed on the NaBH_4_-deposited glass slides. Separate experiments of spraying the fused microdroplets were carried out by fusing the microdroplets containing 100 µM HAuCl_4_ and the microdroplets with no regents and then sprayed on the NaBH_4_-deposited glass slides. The traveling distance of the fused microdroplets were maintained at 15 mm for both sets of experiments. The samples collected on the NaBH_4_-deposited glass slides were transfer to a TEM grid for imaging.

The kinetics of the formation of different nanostructures including individual nanoparticles, aggregates of nanoparticles, and nanowires were measured by increasing the traveling distance of the fused microdroplets. The samples collected on glass slides with different traveling time points of 7.5, 12.1, 21.6, 39.6, and 57.5 µs were transferred to the TEM grid for TEM analysis.

### Characterization of nanostructures

TEM imaging was carried out using FEI Tecnai G2 F20 X-TWIN Transmission Electron Microscope operated at 200 keV and FEI Titan TEM for a high-resolution TEM imaging operated at 300 keV. Three different types of TEM grids made of ultrathin carbon film on lacey carbon support film, carbon film on copper substrate, and carbon on gold substrate (Ted Pella, Redding, CA) were used for TEM imaging. EDS analysis was performed with an EDS detector on the Tecnai TEM. The presence of nanostructures produced in the microdroplets was also examined by dynamic light scattering (DLS). A 100 µL HAuCl_4_solution was sprayed into a 20 mL vial for 30 min. The resultant sample was diluted with 1 mL water used for DLS measurement. The difference in the frequency distribution between the control sample and the nanoparticle solution was measured.

### Mass spectrometry analysis

Thermo Scientific LTQ Orbitrap XL Hybrid Ion Trap-Orbitrap mass spectrometer was used for mass spectrometric analysis. HAuCl_4_ solution at 10 µm concentration prepared in H_2_O was sprayed with high-pressure dry N_2_ gas at 120 psi to generate a stream of microdroplets. No voltage was applied to the solution. The heated capillary inlet of the mass spectrometer was maintained at ~275 °C. The capillary temperature was decreased to 60 °C to examine whether a thermal decomposition of the ions occurred in the capillary. The capillary and tube lens voltages were set as –44 V and –60 V.

Capturing oxygen evolution during nanostructure formation in the microdroplets was carried out by using oxygen-binding molecule tris(2,2′-bipyridyl) dichlororuthenium (II) cation (Ru(bpy)_3_^2+^). Microdroplets containing 10 nM Ru(bpy)_3_^2+^ and 50 µM HAuCl_4_ was sprayed into the mass spectrometer inlet with 100% N_2_ nebulizing gas. As a control experiment, a mass spectrum of the microdroplets containing only 10 nM Ru(bpy)_3_^2+^ was recorded with 100% N_2_ nebulizing gas to ensure that Ru(bpy)_3_^2+^ was not contaminated. To examine whether the oxidation of Ru(bpy)_3_ was induced by oxygen in the air, the nebulization gas was replaced with 100% oxygen gas. The mass spectrum for the samples with Ru(bpy)_3_^2+^ were recorded in positive mode. The heated capillary inlet to the mass spectrometer was maintained at ~275 °C. The capillary and tube lens voltages were set as –44 V and –60 V.

### Data availability

The authors declare that the data supporting the findings of this study are available in this article and its Supplementary Information Files, or from the corresponding authors on request.

## Electronic supplementary material

Supplementary Information
