# Supplementary material for: Spontaneous formation of gold nanostructures in aqueous microdroplets
Source: Nat Commun. 2018 Apr 19;9:1562. doi: 10.1038/s41467-018-04023-z (PMC5908806; doi:10.1038/s41467-018-04023-z)
Supplement: Supplementary file 1 — Supplementary Information [file 41467_2018_4023_MOESM1_ESM.pdf]

Supplementary Information

Spontaneous formation of gold nanostructures in aqueous microdroplets

Jae Kyoo Lee[1], Devleena Samanta[1], Hong Gil Nam[2,3]*, Richard N. Zare[1*]

[1]Department of Chemistry, Stanford University, Stanford, CA 94305, USA.

[2]Center of Plant Aging Research, Institute for Basic Science, Daegu 42988, Republic of Korea.

[3]Department of New Biology, DGIST, Daegu 42988, Republic of Korea.

*Correspondence to: zare@stanford.edu and nam@dgist.ac.kr

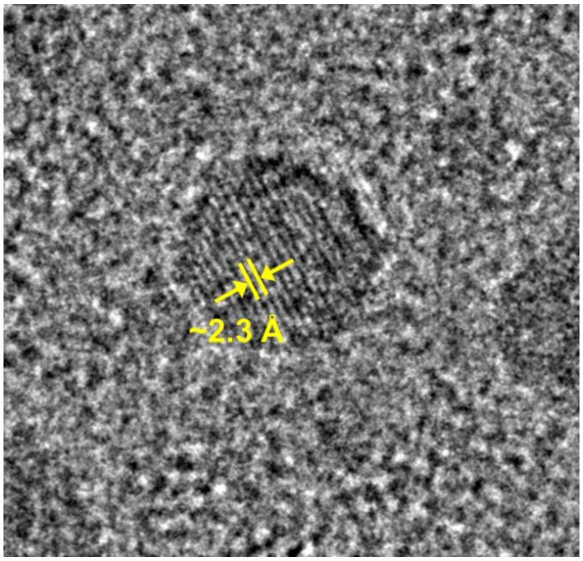

Supplementary Figure 1 | A high-resolution TEM image of AuNP synthesized in fused microdroplets containing the mixture of $HAuCl_4$ and $NaBH_4$ solution. The lattice spacing of the AuNP at ~2.3 Å corresponds to the structure of pure Au (111).

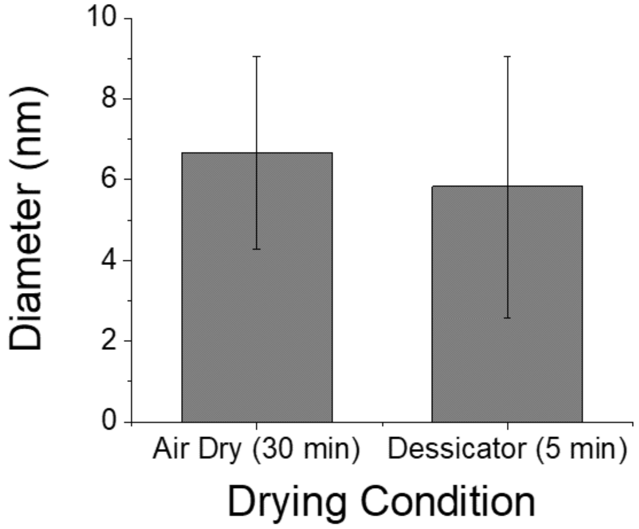

Supplementary Figure 2 | Size difference of AuNPs with different drying condition. The negligible difference of the diameter of AuNP formed in microdroplet and collected on glass slide under different drying conditions demonstrates that the most of the growth occurred in traveling microdroplets rather than in collected liquid on glass slides. Error bar represents standard deviation of three replicates.

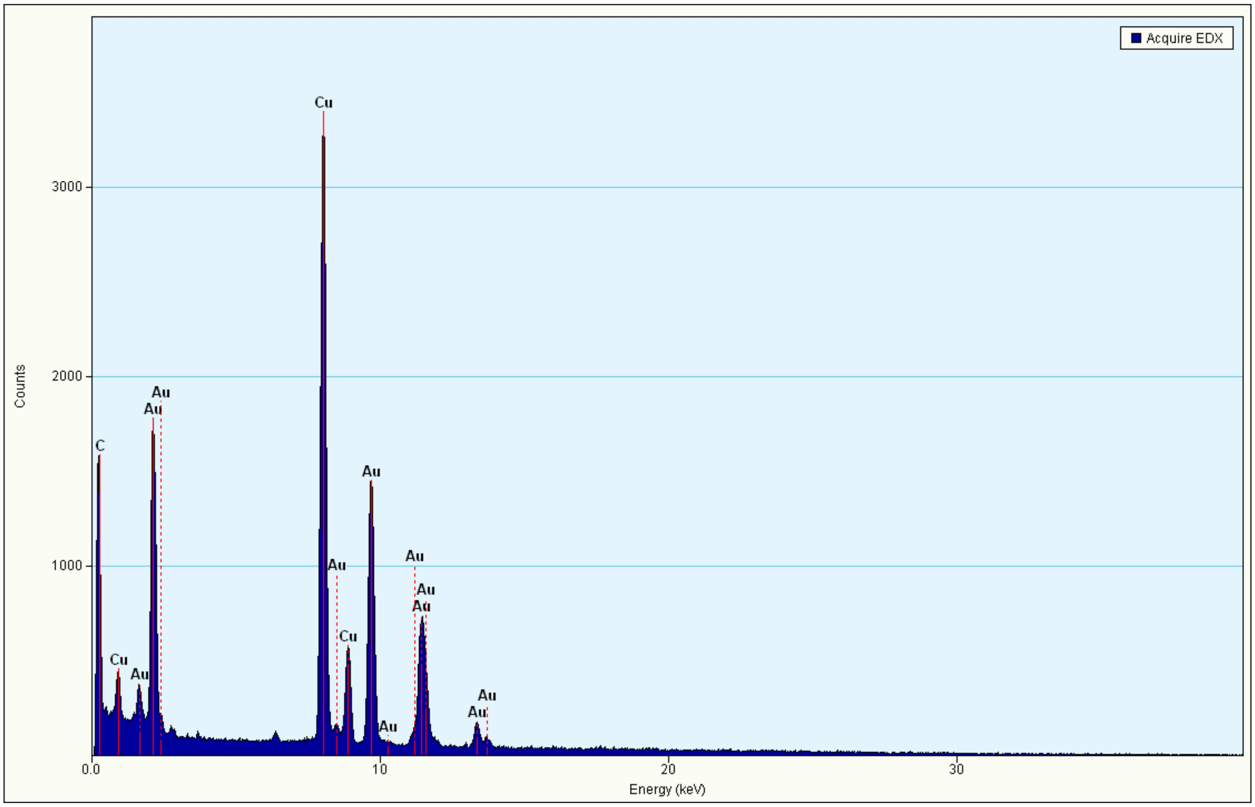

Supplementary Figure 3 | Energy dispersive spectroscopy (EDS) spectrum of AuNPs formed in microdroplets with no externally applied charge.

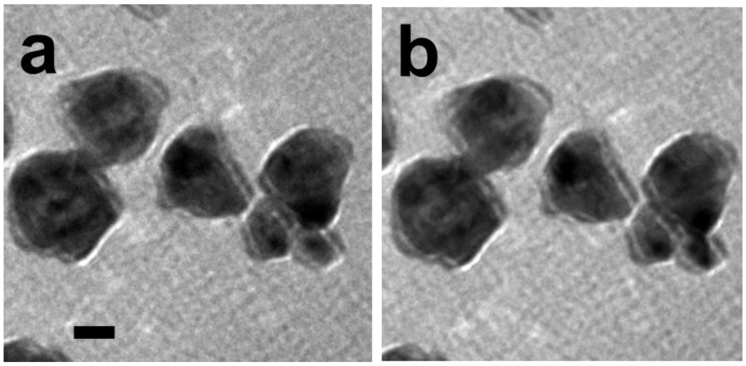

Supplementary Figure 4 | TEM images taken (a) before and (b) after continuous e-beam exposure for 20 minutes. No new formation of nanoparticles as well as sintering or modification of the structure of nanoparticles was observed, indicating that the AuNP formation in microdroplets in the absence of reducing agents was not caused by e-beam irradiation. Scale bar is 10 nm.

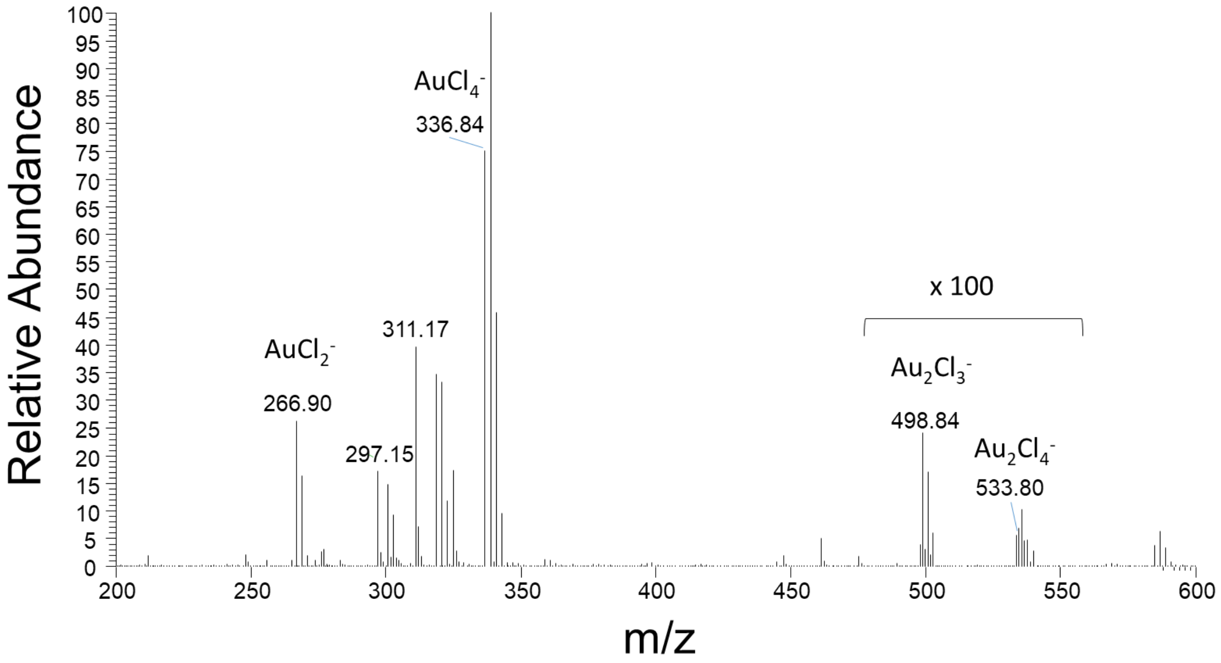

Supplementary Figure 5. Mass spectrum of 10 μM $HAuCl_4$ solution in H_2O sprayed in forms of microdroplets. The *m/z* peaks for both the original $AuCl_4$ (oxidation number +3) and the reduced species, $Au_2Cl_3^-$ (+1), $AuCl_2^-$ (+1), and $Au_2Cl_4^-$ (+1.5) were observed.

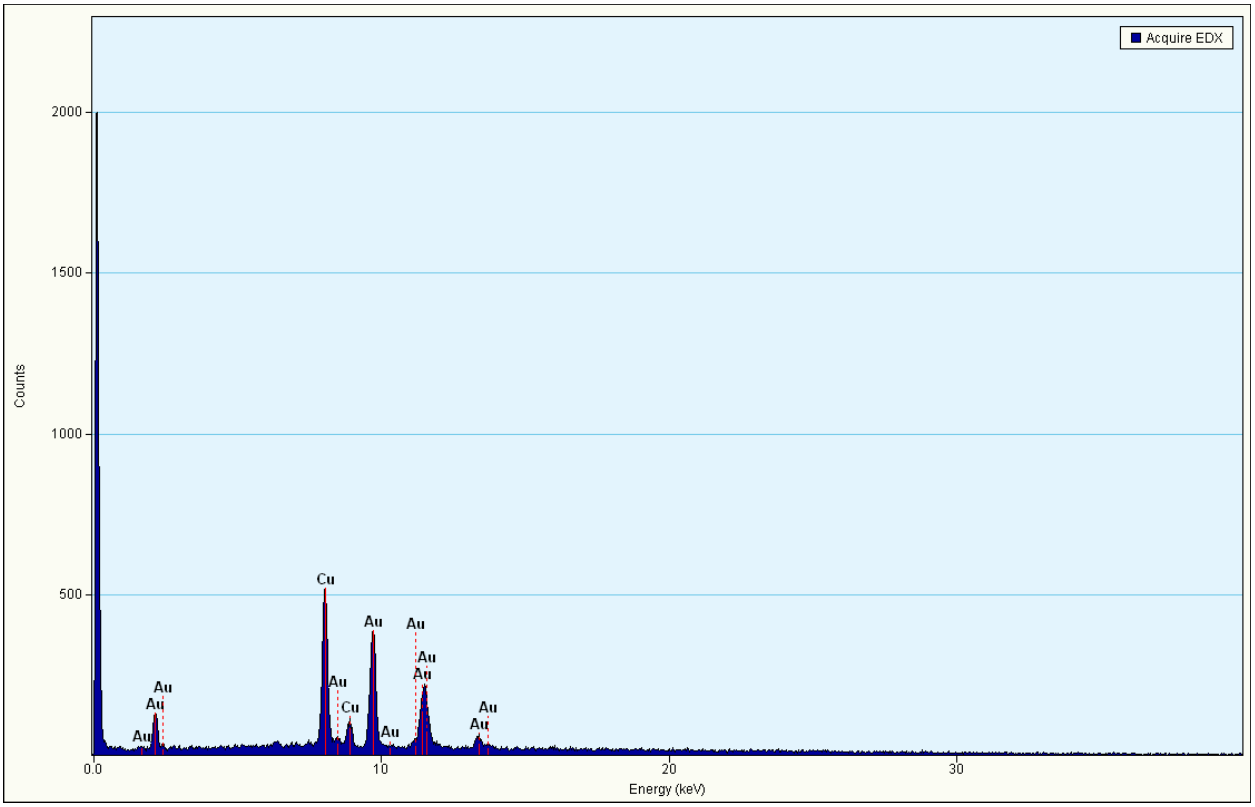

Supplementary Figure 6 | EDS spectrum of AuNWs formed in reducing-agent-free microdroplets.

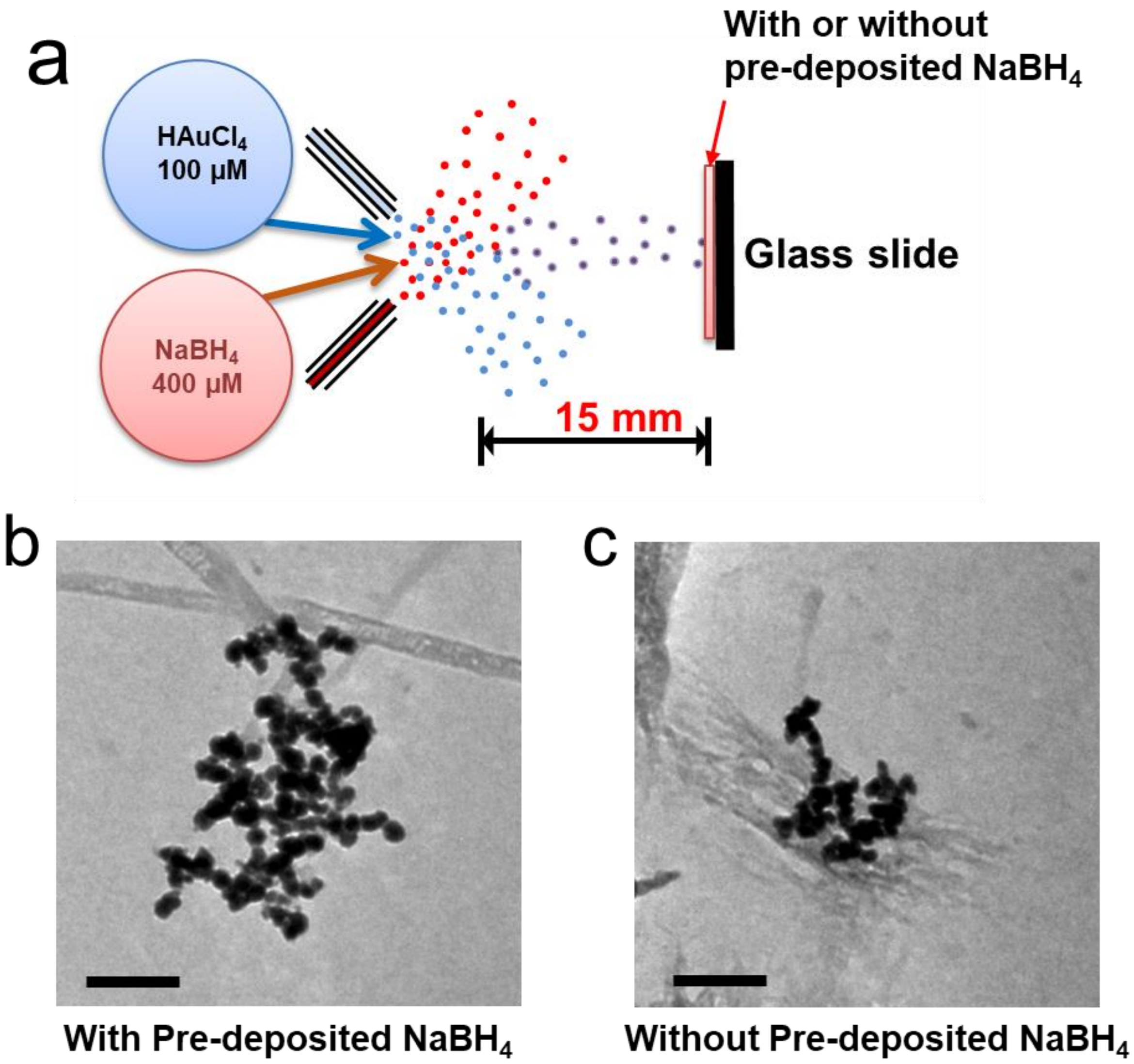

Supplementary Figure 7 | Formation of nanostructures in microdroplet with a collecting slide pre-deposited with and without dried $NaBH_4$. **a**, experiment setup. **b**, TEM image of nanostructures collected on a glass slide with $NaBH_4$ deposited. **c**, TEM image of nanostructures collected on a glass slide with no $NaBH_4$ deposited. Scale bars are 100 nm.

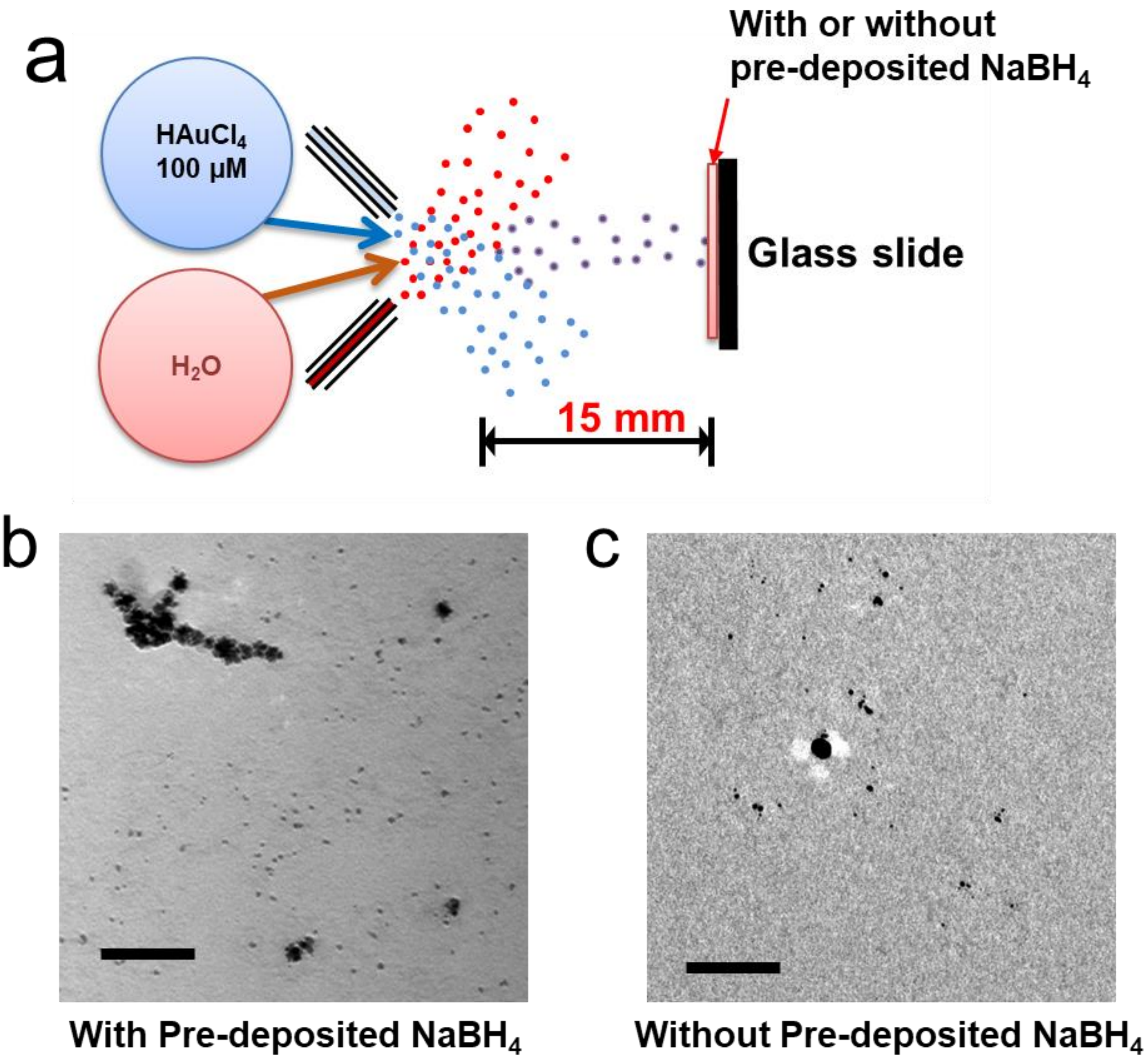

Supplementary Figure 8 | Formation of nanostructures in microdroplet with a collecting slide pre-deposited with and without dried $NaBH_4$. a, experiment setup. **b**, TEM image of nanostructures collected on a glass slide with $NaBH_4$ deposited. **c**, TEM image of nanostructures collected on a glass slide with no $NaBH_4$ deposited. Scale bars are 100 nm.

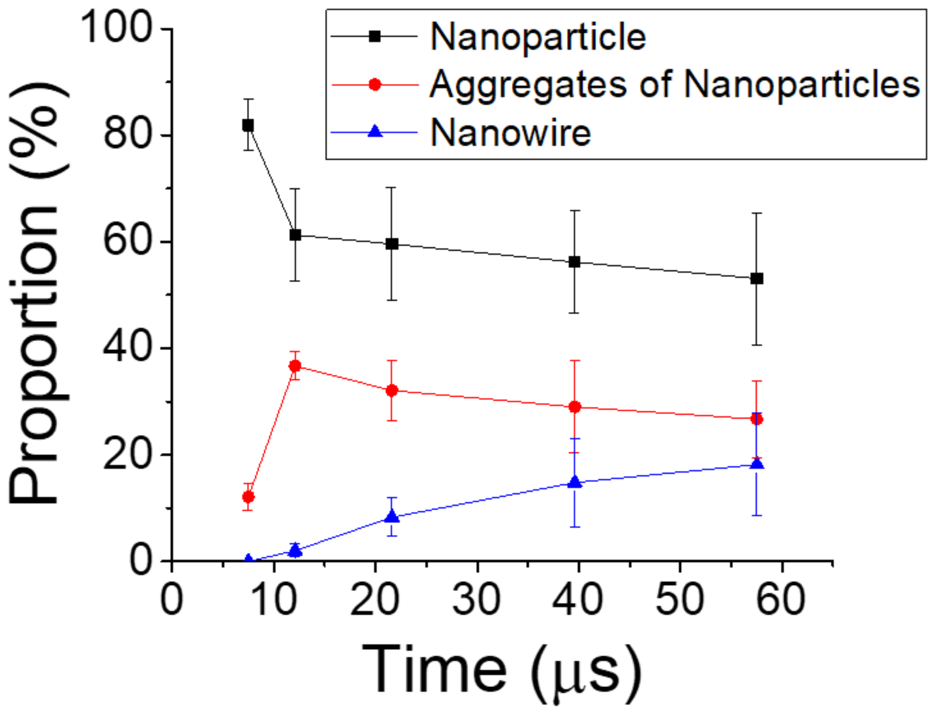

Supplementary Figure 9 | Kinetics of the proportion of different nanostructures formed in reducing-agent-free microdroplets. Error bar represents standard deviation of three replicates.

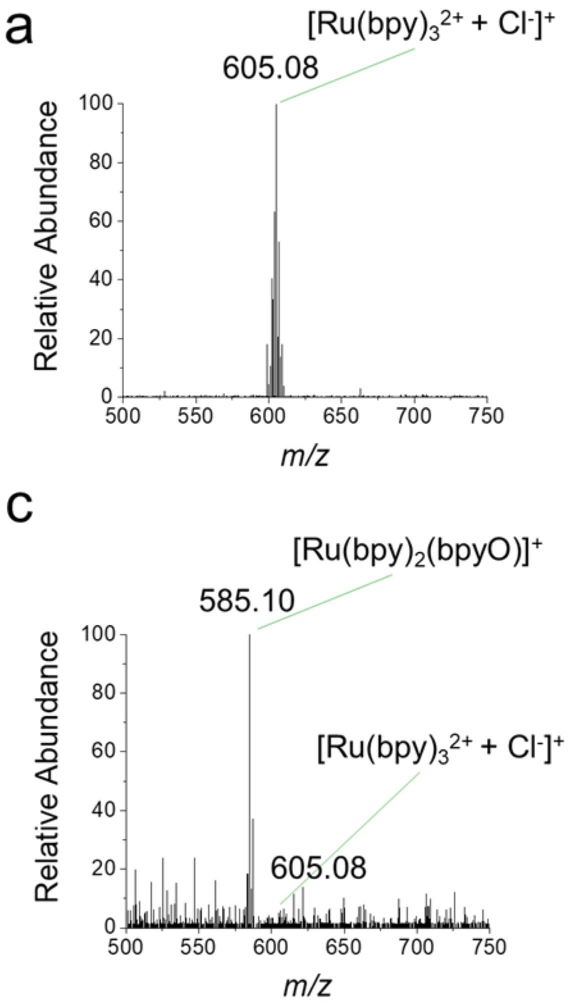

Supplementary Figure 10 | Mass spectra showing oxygen evolution captured during the reduction of gold ions in microdroplets. **a**, Mass spectrum of microdroplets containing 10 nM $Ru(bpy)_3^{2+}$ with 100% N_2 nebulizing gas. m/z 605.08 shows the $Ru(bpy)_3^{2+}$ with Cl^- adduct. **b**, Mass spectrum of microdroplets containing 50 µM $Ru(bpy)_3^{2+}$ with 100% O_2 nebulizing gas. No oxygenated species were observed. **c**, Mass spectrum of microdroplets containing 10 nM $Ru(bpy)_3^{2+}$ and 50 µM $HAuCl_4$ with 100% N_2 nebulizing gas. An oxygenated $Ru(bpy)_3$ species (m/z 585.10) was detected.

Supplementary Table 1. Molecular species observed in 10 µM $HAuCl_4$ solution in H_2O sprayed in forms of microdroplets with a mass spectrometer in negative mode

Observed Molecular Species	Average Oxidation Number	Ion	Observed *m/z*	Theoretical *m/z*	Mass error (ppm)
$Au_2Cl_3^-$	+1	M^-	498.84032	498.83911	2.43
$AuCl_2^-$	+1	M^-	266.90418	266.90371	1.76
$Au_2Cl_4^-$	+1.5	M^-	533.80898	533.80797	1.90
$AuCl_4^-$	+3	M^-	336.84275	336.84141	3.97
